# Supplementary material for: Markov State Models of gene regulatory networks
Source: BMC Syst Biol. 2017 Feb 6;11:14. doi: 10.1186/s12918-017-0394-4 (PMC5294885; doi:10.1186/s12918-017-0394-4)
Supplement: Additional file 1: — Supporting Information. Description of data: Explains models and algorithms used as well as supporting tables and figures. (PDF 5439 kb) [file 12918_2017_394_MOESM1_ESM.pdf]

Supporting Information

1 MISA network reactions

1.0.1 Protein synthesis and degradation reactions

$$A_{01} \xrightarrow{g_0} A_{01} + a$$

$$A_{00} \xrightarrow{g_0} A_{00} + a$$

$$A_{10} \xrightarrow{g_1} A_{10} + a$$

$$A_{11} \xrightarrow{g_0} A_{11} + a$$

$$B_{01} \xrightarrow{g_0} B_{01} + b$$

$$B_{00} \xrightarrow{g_0} B_{00} + b$$

$$B_{10} \xrightarrow{g1} B_{10} + b$$

$$B_{11} \xrightarrow{g0} B_{11} + b$$

$$a \xrightarrow{k} \emptyset$$

$$b \xrightarrow{k} \emptyset$$

1.0.2 Gene Repression

$$A_{00} + 2b \underset{f_r}{\overset{h_r}{\rightleftarrows}} A_{01}$$

$$A_{10} + 2b \underset{f_r}{\overset{h_r}{\rightleftarrows}} A_{11}$$

$$B_{00} + 2a \underset{f_r}{\overset{h_r}{\rightleftarrows}} B_{01}$$

$$B_{10} + 2a \underset{f_r}{\overset{h_r}{\rightleftarrows}} B_{11}$$

1.0.3 Gene Activation

$$A_{00} + 2a \underset{f_a}{\overset{h_a}{\rightleftarrows}} A_{10}$$

$$A_{01} + 2a \underset{f_a}{\overset{h_a}{\rightleftarrows}} A_{11}$$

$$B_{00} + 2b \underset{f_a}{\overset{h_a}{\rightleftarrows}} B_{10}$$

$$B_{01} + 2b \underset{f_a}{\overset{h_a}{\rightleftarrows}} B_{11}$$

2 Toggle switch network reactions

2.0.1 Protein synthesis

$$P_{00} \xrightarrow{g_1} P_{00} + a$$
$$P_{00} \xrightarrow{g_1} P_{00} + b$$
$$P_{01} \xrightarrow{g_0} P_{01} + a$$
$$P_{01} \xrightarrow{g_1} P_{01} + b$$
$$P_{10} \xrightarrow{g_1} P_{10} + a$$
$$P_{10} \xrightarrow{g_0} P_{10} + b$$

2.0.2 Protein degradation

$$a \xrightarrow{k} \emptyset$$
$$b \xrightarrow{k} \emptyset$$

2.0.3 Gene Binding

$$P_{00} + 2a \underset{f}{\overset{h}{\rightleftharpoons}} P_{10}$$
$$P_{00} + 2b \underset{f}{\overset{h}{\rightleftharpoons}} P_{01}$$

3 List of Parameters

Table S1. MISA network parameters

$Figure$	k	g_0	g_1	h_a	h_r	f_a	f_r	τ
1	1	4	16	1e-1	1e-4	1	1e-2	5
2	1	4	16	1e-1	1e-4	1	1e-2	5
3	1	4	16	1e-1	1e-4	1	1e-3,1e-2, 1e-1, 1	5
5	1	4	16	1e-1	1e-4	1	1e-2	5
7	1	4	16	1e-1	1e-4	1	5e-4, 1e-3, 5e-3, 1e-2, 1.5e-2	5
8	1	4	16	1e-3	1e-6	1e-2	1e-4	1

Table S1: The parameters are as follows- k: protein degradation rate, g_0: basal/repressed expression rate, g_1: activated expression rate, h_a: activator binding rate, h_r: repressor binding rate, f_a: activator unbinding rate, f_r: repressor unbinding rate.

Table S2. ETS network parameters

Figure	k	g_0	g_1	h	f	τ
4	1	0	30	1e-6	1e-4	1
6	1	0	40	1e-6,1e-4,1e0,1e2	1e-4,1e-2,1e0,1e2,1e4	1

Table S2: The parameters are as follows- k: protein degradation rate, g_0: basal/repressed expression rate, g_1: activated expression rate, h: protein binding rate, f: protein unbinding rate.

4 Theoretical Background

4.1 Connection between the Master Equation and the Transition Matrix

Herein, we summarize the main previously derived, theoretical results and refer the reader to cited references for further detail. The discrete, Markovian Chemical Master Equation (CME) describes the time-evolution of the probability distribution over the state-space for a well-mixed system of S reacting species with M possible reactions. The state of the system is given by the molecular population vector $\mathbf{x} \in \mathrm{N}^S$. Let $p(\mathbf{x}, t)$ be the probability that the system is found in the state $\mathbf{x}$ at time t, and let $a_\mu(\mathbf{x})dt$ and $\boldsymbol{\nu_\mu}$ be the propensity function and stoichiometric transition vector, respectively, of the possible reactions $\mu = (1, 2, ..., M)$. Then the CME is given by

$$\frac{\partial p(\mathbf{x}, t|\mathbf{x}_0, t_0)}{\partial t} = \sum_{\mu}^{M} [a_\mu(\mathbf{x} - \boldsymbol{\nu_\mu}) p(\mathbf{x} - \boldsymbol{\nu_\mu}, t|\mathbf{x}_0, t_0) - a_\mu(\mathbf{x}, t|\mathbf{x}_0, t_0)]. \quad (1)$$

In vector-matrix form,

$$\frac{d\mathbf{p}(\mathbf{X}, t)}{dt} = \mathbf{K}\mathbf{p}(\mathbf{X}, t), \quad (2)$$

where the off-diagonal element K_{ij} gives the time-independent rate of transitioning from state $\mathbf{x}_i$ to state $\mathbf{x}_j$, and $K_{ii} = -\sum_{j \neq i} K_{ji}$ (i.e., columns sum to 0). Given the probability at time t, $\mathbf{p}(\mathbf{X}, t)$, the probability density at a later time (so-called lag-time) τ may be computed by:

$$\mathbf{p}(\mathbf{X}, \tau + t) = \exp(\mathbf{K}\tau)\mathbf{p}(\mathbf{X}, t) \quad (3)$$

where $\exp(\mathbf{K}\tau)$ is the matrix-exponential of $\mathbf{K}\tau$. Many biochemical reaction networks are modeled as open systems, such that the molecular state space is technically infinite. That is, a given molecule in the system may occur with with any positive integer value for its copy number, though practically only a finite number of states are reachable in finite time. We assume here that the CME is approximated over a finite subspace, e.g., via the Finite State Projection Algorithm [1].

The linear system may be recast using the row-stochastic transition matrix [2]

$$\mathbf{T}(\tau) = \exp(\mathbf{K}^T \tau) \quad (4)$$

where the off-diagonal elements $T_{ij}(\tau)$ give the probability that, given the system is in state i (corresponding to molecular population vector $\mathbf{x}_i$), it will then be found in state j after lag time τ. Probability is preserved, and thus rows sum to 1. The relationship between the state reaction matrix $\mathbf{K}$ and the stochastic transition matrix $\mathbf{T}$ leads to the relationship for the implied timescales:

$$t_i = \frac{-\tau}{\ln \lambda_i(\tau)}. \quad (5)$$

The dynamics is propagated according to the Chapman-Kolmogorov equation, which gives the probability at discrete times $t + k\tau$, with $k = \{1, 2, 3, ...\}$ by

$$\mathbf{p}^T(\mathbf{X}, k\tau + t) = \mathbf{p}^T(\mathbf{X}, t)[\mathbf{T}(\tau)]^k \quad (6)$$

.

4.2 Building Markov State Models with PCCA+

The goal of the clustering approach is to derive a new coarse-grained stochastic transition matrix with drastically reduced dimensionality relative to to the original matrix $\mathbf{T}$, and which preserves as accurately as possible the slow system dynamics. Consider a reactive system in which $\mathbf{T}$ is defined over a total number of reachable states N (i.e., if a system of S molecular species has a maximum copy number per species of n, then $N \sim n^S$). Then the Perron eigenvalue $\lambda_1 = 1$ corresponds to the left-eigenvector π according to

$$\pi^T \mathbf{T} = \pi^T \tag{7}$$

that is, $\pi^T = (\pi_1, ..., \pi_N)$ gives the stationary probability over N states. If the reactive system has the property of metastability, then the dynamics can be approximately decomposed into fast and slow processes, with fast transitions occuring within metastable states, and slow transitions carrying the system between metastable states. For sufficient separation of fast and slow timescales, Markovian hopping between metastable states is a good model of global dynamics, and the full dynamics may be projected onto this slow subspace. In this case, for a sufficient ordering of the N states, $\mathbf{T}$ has a dominant block-diagonal structure with some number C of weakly coupled blocks. In such systems, a cluster of C eigenvalues can be found near the Perron root, with $\lambda_1 \geq \lambda_i \geq \lambda_C$ corresponding to the slow processes in the system, and all fast processes corresponding to rapidly decaying processes with eigenvalues $\lambda_i < \lambda_C$. The PCCA+ algorithm [3] determines membership vectors χ which assign states $i \in \{1, ..., N\}$ to clusters of states $j \in \{1, ..., C\}$ (with $C < N$) where grades of membership are given by $\chi_j(i) \in [0, 1]$. (The original PCCA algorithm was based on strict "crisp" assignments $\chi_j(i) \in \{0, 1\}$, but the algorithm was not robust numerically). The membership vectors satisfy the linear transformation

$$\chi = \psi \mathbf{B} \tag{8}$$

where $\psi = [\psi_1, ..., \psi_C]$ is the $N \times C$ matrix constructed from the C dominant right-eigenvectors, and $\mathbf{B}$ is a non-singular matrix whose elements are determined by an unconstrained optimization procedure. The membership vectors determine a projection of the full $N \times N$ transition matrix $\mathbf{T}$ onto a reduced (coarse) subspace, and we denote this reduced $C \times C$ transition matrix as $\tilde{\mathbf{T}}$. As discussed previously, the optimization procedure can be be based on maximizing the metastability, taken to be measured by trace($\tilde{\mathbf{T}}$).

The coarse-grained stationary probability is given by the projection

$$\tilde{\pi}^T = \pi^T \chi, \tag{9}$$

and the transition probabilities in the reduced space are given by

$$\tilde{T}_{ii}(\tau) = \frac{\langle \chi_i, \mathbf{T}(\tau) \chi_i \rangle_\pi}{\tilde{\pi}_i}, \tag{10}$$

(for the probability to remain in state i within time τ) and

$$\tilde{T}_{ij}(\tau) = \frac{\langle \chi_i, \mathbf{T}(\tau) \chi_j \rangle_\pi}{\tilde{\pi}_i}, \tag{11}$$

(for the probabilities to jump from state i to state j in τ, where $\langle u, v \rangle_\pi$ is the π-weighted inner product of u and v). Therefore we may write

$$\tilde{\mathbf{T}}(\tau) = \tilde{D} \langle \chi_i, \mathbf{T}(\tau) \chi_j \rangle_\pi \tag{12}$$

where $\tilde{D} = \text{diag}(\tilde{\pi}_1, ..., \tilde{\pi}_C)$, or equivalently,

$$\tilde{\mathbf{T}}(\tau) = \left(\chi^T D \chi\right)^{-1} \chi^T D \mathbf{T}(\tau) \chi \tag{13}$$

where $D = \text{diag}(\pi_1, ..., \pi_N)$.

One may compare, on the coarse sub-space, the true system dynamics given by $\mathbf{T}$ with the dynamics given by the reduced Markov State Model, $\tilde{\mathbf{T}}$. Given an initial probability vector $\mathbf{p}(\mathbf{x}, t_0)$, the true dynamics would propagate the system according to $\mathbf{p}^T(\mathbf{x}, k\tau + t_0) = \mathbf{p}^T(\mathbf{x}, t_0)\mathbf{T}(\tau)^k$. By projection,

$$\tilde{\mathbf{p}}^T_{\text{true}}(\mathbf{x}, k\tau + t_0) = [\mathbf{p}^T(\mathbf{x}, t_0)[\mathbf{T}(\tau)]^k]\chi \tag{14}$$

and

$$\tilde{\mathbf{p}}^T_{\text{MSM}}(\mathbf{x}, k\tau + t_0) = [\mathbf{p}^T(\mathbf{x}, t_0)\chi][\tilde{\mathbf{T}}(\tau)]^k. \tag{15}$$

The error after k timesteps is thus given by: $\epsilon(k) = ||\mathbf{p}^T(\mathbf{x}, t_0)\left([\mathbf{T}(\tau)]^k\chi - \chi[\tilde{\mathbf{T}}(\tau)]^k\right)||_2$. As discussed previously in [2] the maximum error associated with the projection of $\mathbf{T}$ onto $\tilde{\mathbf{T}}$ in the reduced space (independent of initial state) is given by:

$$\epsilon(k) = ||[\mathbf{T}(\tau)]^k\chi - \chi[\tilde{\mathbf{T}}(\tau)]^k||_2 \tag{16}$$

4.3 Transition Path Theory

Transition path theory[4] is used in this study to extract information about the kinetics and mechanism of a transition between a starting set of states A and ending set of states B. First, we calculate the flux between states during the transition between A and B according to (17). We obtain a net flux from (18) that extracts only the flux that contributes to the transition. Finally in (19), flux of each pathway can be used to compute the probability of traversing the pathway. Equation (20) and (21) show how to reduce the full transition matrix to a reduced representation if it is desirable to perform the pathway probability calculation on the metastable states of the system.

To compute the probabilities of the transition paths, from a starting state A to an ending state B, one must calculate the effective flux f_{ij} for all pairs of states. The transition probability relevant to the A to B state transition is found by taking the product of forward commitor q_i^-, transition probability T_{ij}, and backward commitor q_j^+. The forwards and backwards commitors are probablities of transitioning to B or returning to A respectively. Finally, the flux is obtained by weighting it by its starting state stationary probability.

$$f_{ij} = \pi_i q_i^- T_{ij} q_j^+ \tag{17}$$

To get the net flow from A to B, only positive fluxes are considered by taking differences of the fluxes between pairs of states.

$$f_{ij}^+ = max\{0, f_{ij} - f_{ji}\} \tag{18}$$

After extracting out the pathways by removing their fluxes sequentially from the system, the probability of each pathway is calculated by taking the ratio of the flux of the individual path to the total flux of all paths.

$$p_i = \frac{f_i}{\sum_j f_j} \tag{19}$$

The total stationary probability $\tilde{\pi_i}$ of a cluster of states is simply the sum of its individual states π_i in the cluster.

$$\tilde{\pi_i} = \sum_{i \in I} \pi_i \tag{20}$$

The transition probablities between clusters $\tilde{T_{IJ}}$ is found by summing transition probabilities between states T_{ij} weighted by their stationary probabilities of the states π_i and normalized by the stationary probabilities of the clusters π_I.

$$\tilde{T_{IJ}} = \frac{1}{\pi_I} \sum_{i \in I} \pi_i \sum_{j \in J} T_{ij} \tag{21}$$

5 Pseudocode

Step 1. Define propensity functions and stoichiometric transition vectors for all possible reactions in network model (see Section 4.1, equation (1)) .
Step2. Enumerate a reaction matrix in terms of the propensity functions stoichiometric transition vectors (see Section 4.1, equation (2)).
Step3. Take the matrix exponential of the reaction matrix to obtain a transition matrix (see Section 4.1, equation (4)).
Step 4. Calculate the left/right eigenvectors and eigenvalues (see Section 4.2) .
Step 5.Use PCCA+ algorithm, to solve for macrostate to microstate mapping using the transition matrix, eigenvalues, and eigenvectors (see Section 4.2).
Step 6. Coarse-grain the transition matrix using the PCCA+ mapping into a Markov State Model of the core states (see Section 4.2, equation (13)).
Step 7. Analysis of global dynamics of network model in terms of core states defined by Markov State Model

- Apply pathway decomposition , to obtain a probability distribution of possible paths in terms of core states (see Section 4.3).
- Propagate the dynamics of the core states by simulating the Markov State Model (see Section 4.3, equation (15)).

6 Supporting Figures

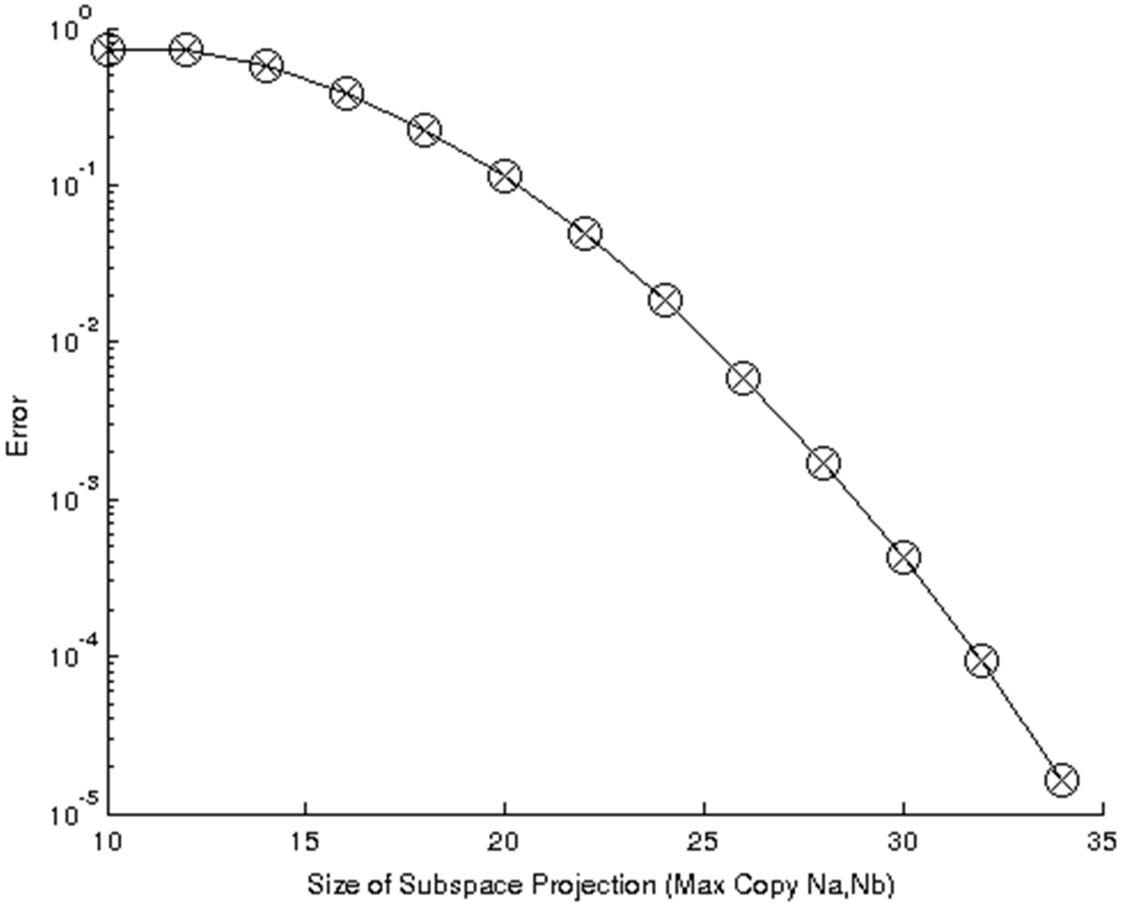

Figure S1: MISA network, Parameter Set 1. The truncation error as a function of the size of the subspace projection after 1000 time steps (approximately the relaxation time). The x and o markers denote two randomly initialized state vectors. The two curves virtually overlap , showing that system has lost memory of its initial starting point. The chosen subspace for all parameter sets are cut off at N = 30 proteins , while the maximum gene expression rate (g1/k) is 16.

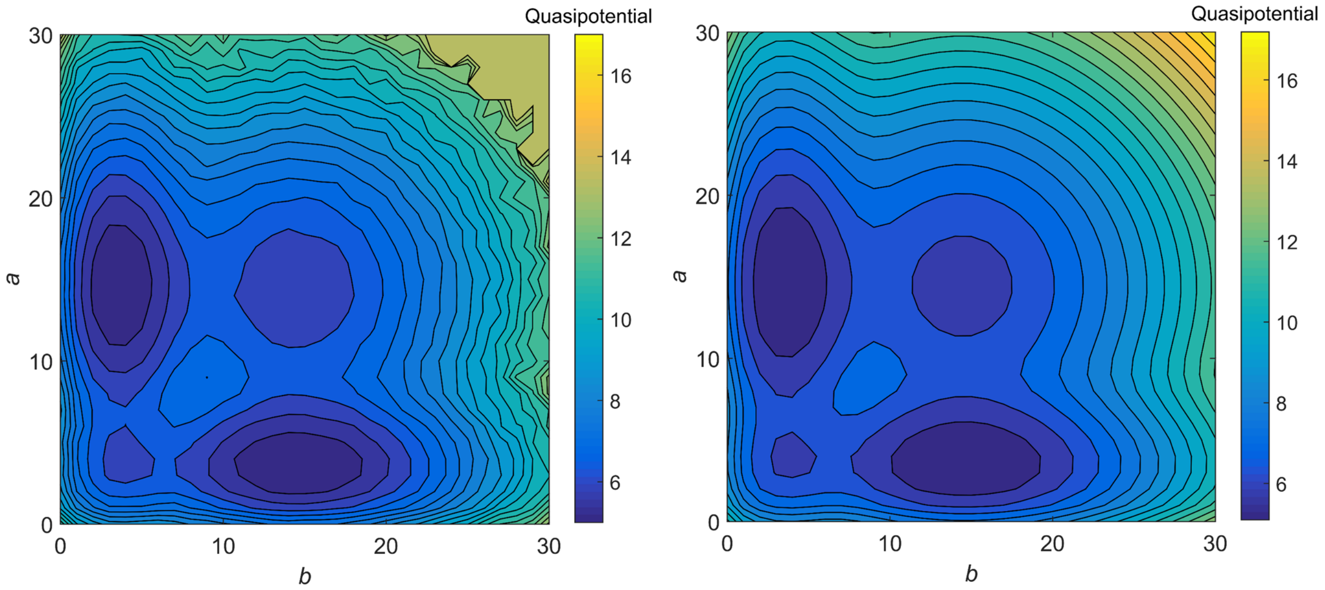

Figure S2: MISA network, Paramater Set 1. Brute Force SSA simulation (left) vs Chemical Master Equation (right) potential landscape comparison

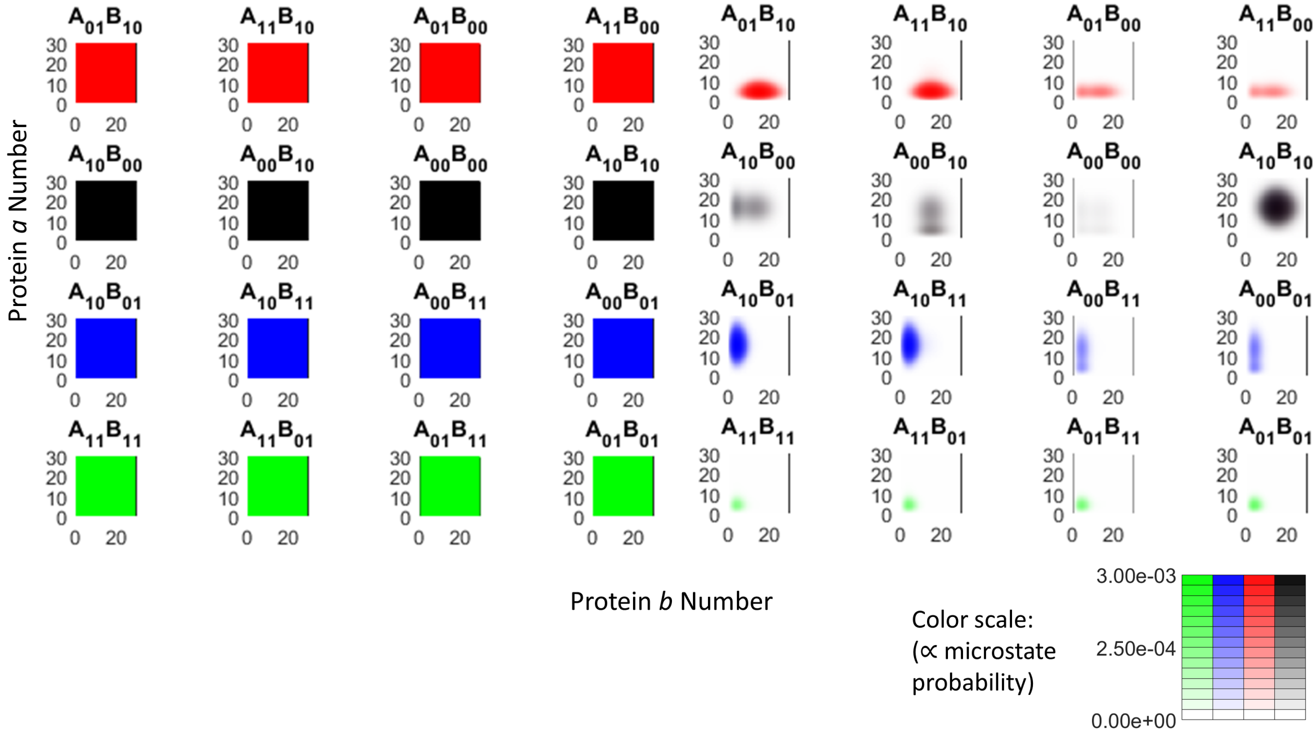

Figure S3: MISA network, Parameter Set 1, detailed microstate to macrostate mapping (see Fig. 2). (Left) The entirety of the enumerated state-space (all N=15,376 (31x31x4x4) microstates) is shown, and each microstate is colored according to its macrostate assignment, as determined by the PCCA+ crisp partitioning. Each of the sixteen panels corresponds to a particular DNA promoter binding configuration A_{00}, etc., as defined in SI I, MISA reactions, and Fig. 2B. For this parameter set, the promoter configuration determined the macrostate assignment exactly. (Right) The color-scale for each microstate is proportional to steady-state probability, such that low-probability-density regions appear white. Each of the promoter configurations shows a distinct pattern of protein expression. Grouping the high-density microstates within a macrostate and projecting onto the protein subspace gives rise to the ellipsoids shown in Fig. 2A. The probability density contributed by each gene state to each macrostate is shown in Table S3.

4 State MSM Gene Compositions

Macrostate	Gene	Composition	Macrostate	Gene	Composition
0	$A_{01}B_{10}$	47.9%	2	$A_{10}B_{01}$	47.9%
0	$A_{11}B_{10}$	39.9%	2	$A_{10}B_{11}$	39.9%
0	$A_{01}B_{00}$	6.7%	2	$A_{00}B_{11}$	5.5%
0	$A_{11}B_{00}$	5.5%	2	$A_{00}B_{01}$	6.7%
1	$A_{10}B_{00}$	1.8%	3	$A_{11}B_{11}$	20.8%
1	$A_{00}B_{10}$	11.8%	3	$A_{11}B_{01}$	24.8%
1	$A_{00}B_{00}$	11.8%	3	$A_{01}B_{11}$	24.8%
1	$A_{10}B_{10}$	74.6%	3	$A_{01}B_{01}$	29.7%

Table S3. MISA network, Parameter set 1, Contribution of each promoter configuration to total probability density of each macrostate. Each macrostate contains four promoter configurations. The probability contribution of each configuration to the macrostate (composition) is shown. That is, within each macrostate the probabilities sum to 100%. While more than one configuration contributes significantly to the probability density within a macrostate, the configurations contributing the most probability to the macrostate are termed 'representative' and shown schematically in Figure 2B.

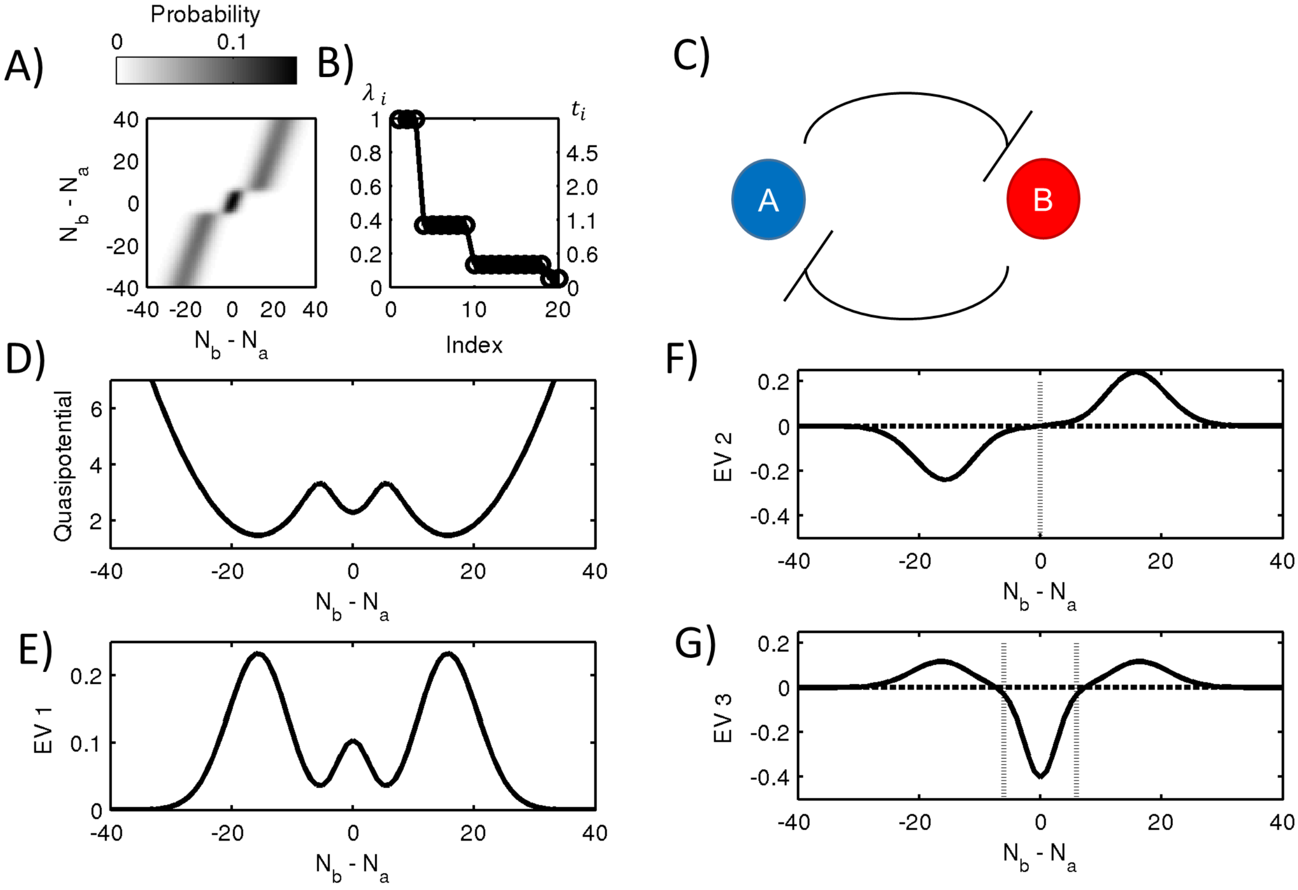

Figure S4: ETS network, Parameter Set 1. (A) Transition matrix in terms of number of proteins B (N_b) - number of proteins A (N_a)), where probability is showed by the darkness intensity (B) The first twenty eigenvalues in descending order (C) Schematic of exclusive toggle switch (D) The one-dimensional quasipotential in terms of N_b - N_a (E) The first eigenvector in terms of in terms of N_b - N_a (F) The second eigenvector in terms of in terms of N_b - N_a (G) The third eigenvector in terms of in terms of N_b - N_a

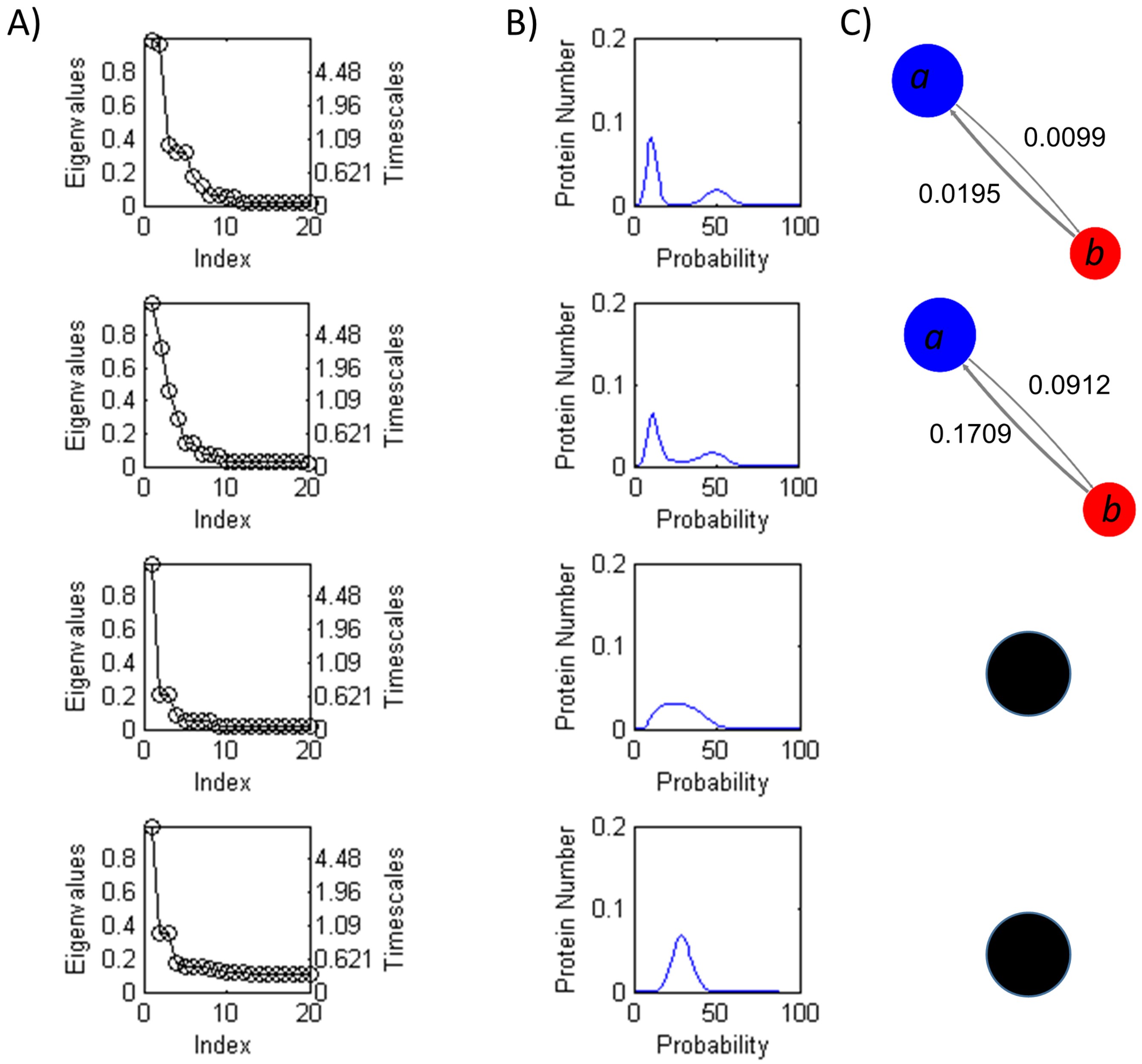

Figure S5: Dependence of the Self Regulating Single Gene Network eigenvalues, probability distributions, and state transition graphs on the binding parameter f. The model is defined as in [5], with parameters $g_1 = 50$, $g_0 = 10$, $X_{eq} = 25$, $h = f/X_{eq}$, $k = 1$. Top to Bottom: increasing f = 0.01, 0.1, 1, 10 in units of protein degradation rate, k (A) The eigenvalue spectrum of $\mathbf{T}(\tau)$ for $\tau = 1$, and associated timescales (B) The steady-state probability distribution, calculated from the CME truncated to $n < 100$, where n is the copy-number of the expressed gene. (C) The state transition graph produced by applying the Markov State Model framework. A timescale threshold of $t = 2$ was used to determine the number of metastable macrostates to retain in the MSM (in time-units of k^{-1}, the inverse protein degradation rate). For $f = 0.01$ and $f = 0.1$, the eigenvalue spectrum reveals a slow system timescales $t_2 = 33.17$ and $t_2 = 3.05$, respectively, thus two macrostates are retained in the MSM (edge labels give the transition probabilities within $\tau = 1$). These macrostates correspond directly to the gene off and gene on states (a and b, respectively). For $f = 1$ and $f = 10$, the eigenvalue spectrum shows some structure, but all implied timescales are shorter than $t = 2$, thus one macrostate is retained in the MSM.

Table S4. Estimated rate constants from the two-state MSMs for the self-regulating single-gene network. Longest system timescale implied by the eigenvalue spectrum of T, and T_s, the switching time estimated from model rate parameters based on [6].

$f(k)$	$k_{ab}(k)$ (from MSM)	$k_{ba}(k)$ (from MSM)	$t_2(k)$	T_s (switching time, units k) [6]
0.01	0.0101	0.0198	33.17	33.34
0.1	0.1058	0.1982	3.05	3.34

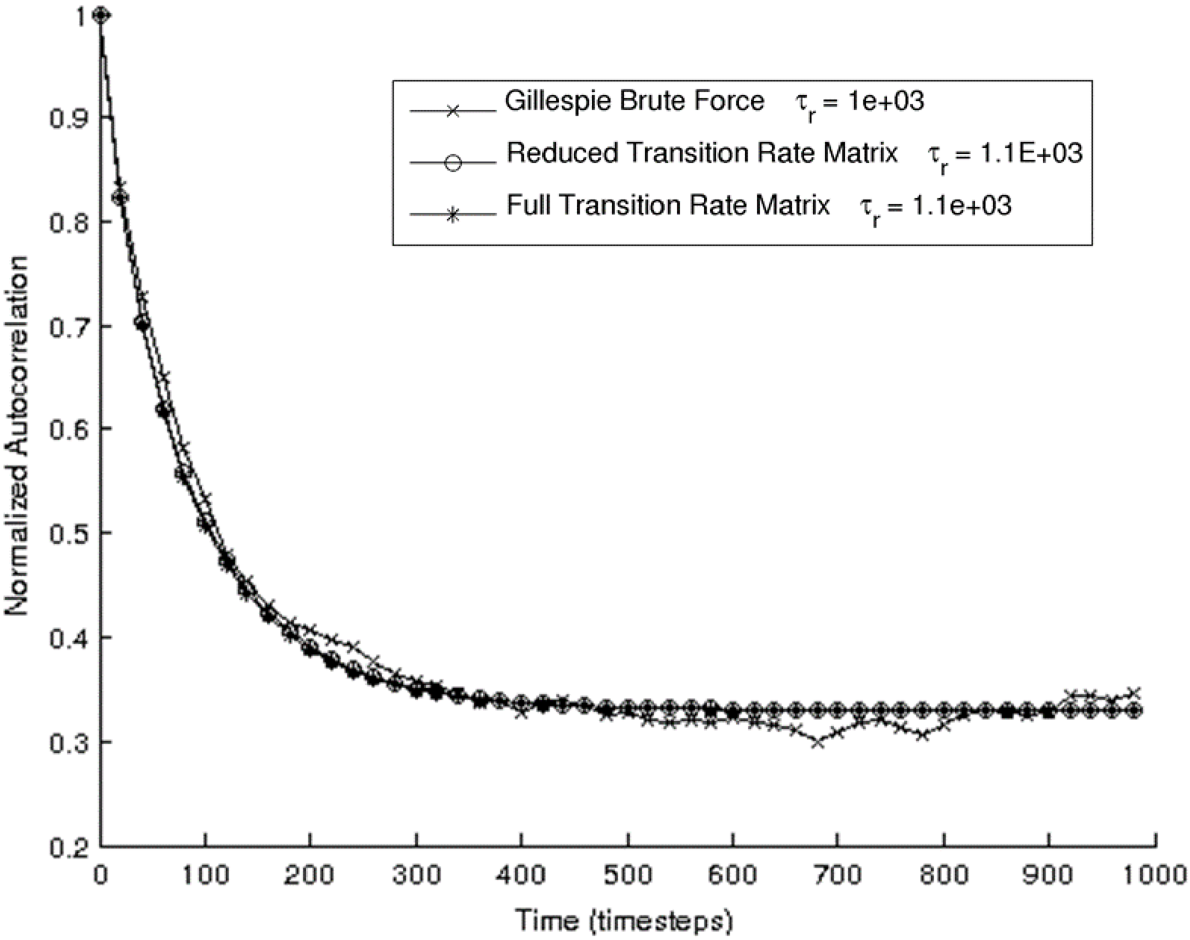

Figure S6: MISA network, Parameter Set 1. Autocorrelation function for the polarized state calculated from a simulation trajectory using Gilespie Brute Force, Reduced Transition Rate Matrix, and Full Transition Rate Matrix. Relaxation constants, τ_r, are calculated from fitting the curve to single decaying exponential.

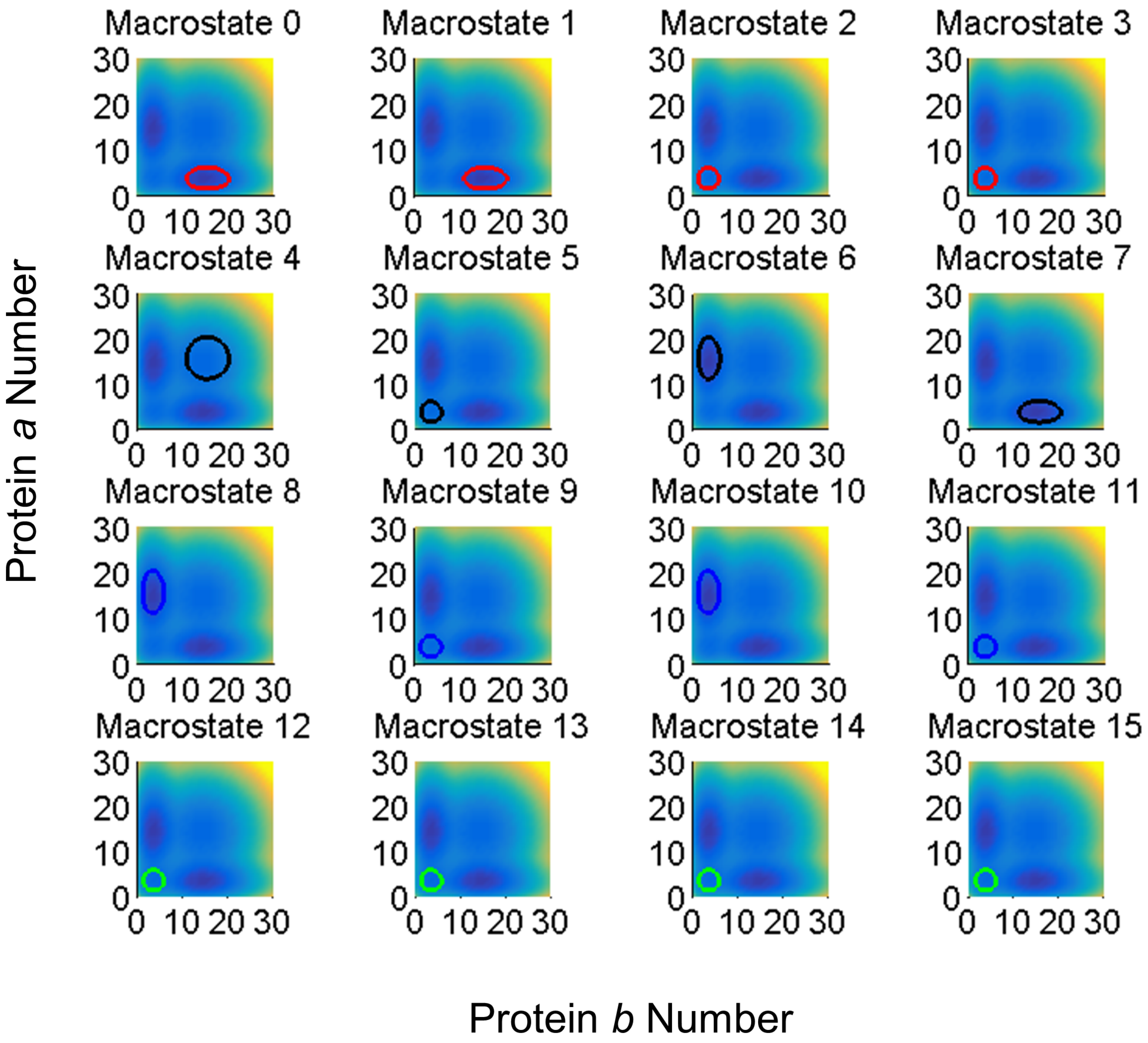

Figure S7: MISA network, Parameter Set 8. 16-macrostate MSM: 50% probability contours superimposed onto the dominant eigenvalues. The index of each macrostate is labeled above each plot.

16 State MSM Gene Compositions

Macrostate	Gene	Composition	Macrostate	Gene	Composition
0	$A_{01}B_{10}$	100%	8	$A_{10}B_{01}$	100%
1	$A_{11}B_{10}$	100%	9	$A_{10}B_{11}$	100%
2	$A_{01}B_{00}$	100%	10	$A_{00}B_{11}$	100%
3	$A_{11}B_{00}$	100%	11	$A_{00}B_{01}$	100%
4	$A_{10}B_{00}$	100%	12	$A_{11}B_{11}$	100%
5	$A_{00}B_{10}$	100%	13	$A_{11}B_{01}$	100%
6	$A_{00}B_{00}$	100%	14	$A_{01}B_{11}$	100%
7	$A_{10}B_{10}$	100%	15	$A_{01}B_{01}$	100%

4 State MSM Gene Compositions

Macrostate	Gene	Composition	Macrostate	Gene	Composition
0	$A_{01}B_{10}$	26.1%	2	$A_{10}B_{01}$	26.1%
0	$A_{11}B_{10}$	20.9%	2	$A_{10}B_{11}$	20.9%
0	$A_{01}B_{00}$	29.4%	2	$A_{00}B_{11}$	23.5%
0	$A_{11}B_{00}$	23.5%	2	$A_{00}B_{01}$	29.4%
1	$A_{10}B_{00}$	24.9%	3	$A_{11}B_{11}$	19.8%
1	$A_{00}B_{10}$	24.9%	3	$A_{11}B_{01}$	24.7%
1	$A_{00}B_{00}$	28.5%	3	$A_{01}B_{11}$	24.7%
1	$A_{10}B_{10}$	21.7%	3	$A_{01}B_{01}$	30.8%

Table S5. MISA network, Parameter Set 8. Gene composition for every state in the 16 state MSM (top) and 4 state MSM (bottom), where color corresponds to its respective substate. Compositions associated with each gene configuration is defined as the percentage of the total stationary probability attributed to that state.

7 List of Pathways of 16 State MSM

Table S6. Paths from State 1 to 11

State Path	Probability (%)
1,5,6,10,11	24
1,3,6,10,11	21
1,0,7,4,8,9,11	18.5
1,3,2,4,8,10,11	9.2
1,0,2,4,8,9,11	8.7
1,5,11	3.3
1,0,7,9,11	2.8
1,15,11	2.2
1,0,7,4,8,10,11	1.9
1,0,14,9,11	1.8
1,3,13,10,11	1.7
1,0,2,13,8,9,11	1.4

Table S7. Paths from State 11 to 1

State Path	Probability (%)
11,5,7,0,1	23.2
11,9,7,0,1	21.7
11,10,6,4,2,3,1	20.6
11,9,8,4,2,0,1	8.3
11,10,8,4,2,3,1	7.8
11,5,1	3.1
11,10,6,3,1	2.7
11,15,1	2.2
11,10,13,3,1	1.8
11,9,14,0,1	1.7
11,10,8,4,2,0,1	1.5
11,9,8,12,2,0,1	1.1

8 References

1. Munsky, Brian, and Mustafa Khammash. "The finite state projection algorithm for the solution of the chemical master equation." The Journal of chemical physics 124.4 (2006): 044104.

2. Prinz, Jan-Hendrik, et al. "Markov models of molecular kinetics: Generation and validation." The Journal of chemical physics 134.17 (2011): 174105.

3. Deuflhard, Peter, and Marcus Weber. "Robust Perron cluster analysis in conformation dynamics." Linear algebra and its applications 398 (2005): 161-184.

4. Noe, Frank, et al. "Constructing the equilibrium ensemble of folding pathways from short off-equilibrium simulations." Proceedings of the National Academy of Sciences 106.45 (2009): 19011-19016.

5. Schultz, Daniel, Jos N. Onuchic, and Peter G. Wolynes. "Understanding stochastic simulations of the smallest genetic networks." The Journal of chemical physics 126.24 (2007): 245102.

6. Ramos, A. F., G. C. P. Innocentini, and J. E. M. Hornos. "Exact time-dependent solutions for a self-regulating gene." Physical Review E 83.6 (2011): 062902.
